# Trends in Deaths of Despair Among Older Adults, 1999-2018

**DOI:** 10.1093/geroni/igaa057.362

**Published:** 2020-12-16

**Authors:** Peter Sun, Edward Lawlor, Nancy Morrow-Howell, Sojung Park, Timothy McBride

**Affiliations:** 1 Washington University in St. Louis, Saint Louis, Missouri, United States; 2 Washington University in St. Louis, St. Louis, Missouri, United States

## Abstract

“Deaths of despair” (DOD) is a new phenomenon that includes deaths attributable to suicides, drug overdoses, and alcohol-related liver diseases. While many studies have focused on the rise of DOD in midlife, there is little research that focuses on the trends of DOD in later life. To address this gap, we used publicly available data from the Centers for Disease Control and Prevention to carry out descriptive and spatial analyses of DOD among older adults (aged 65 and over) from 1999 to 2018. Nationwide, we found that DOD rates of older adults increased from 2009 (44.7 deaths per 100,000) to 2018 (57.4). This increase was notable among male older adults who were Black non-Hispanics and White non-Hispanics (WNH); however, Hispanic populations saw a 4.8% decrease in DOD between 1999 and 2018. Of the WNH decedents in 2018, alcohol-related liver diseases were the most common cause of death (55%), followed by suicides (36%) and drug overdoses (9%). Additionally, 55% had a high school degree or less, 22% had some college but no bachelor’s degree (BA), and 22% had a BA or more. Geographically, we found that DOD was concentrated in the West in 1999 but has since then spread nationwide. In 2018, the highest rates of DOD among WNH were in the western and southern states. These findings suggest that attention to deaths of despair cannot be limited to midlife; policies and program interventions aimed at slowing this epidemic must include people in later life as well.

